# Examining Longitudinal, Reciprocal Relations Between Mental Health Service Use and Mental Health Symptoms

**DOI:** 10.1007/s11414-025-09963-1

**Published:** 2025-08-26

**Authors:** Jennifer Murphy, Youngmi Kim, Kyeongmo Kim, Tasha Pelletier, Kristen Kerr

**Affiliations:** 1 School of Social Work, University of Texas at Arlington, Arlington, TX 76010 USA; 2 School of Social Work, Virginia Commonwealth University, 1000 Floyd Avenue, Richmond, PO Box 842027, 23220 USA

## Abstract

Adolescence is often identified as the period of time when individuals first begin to experience mental health needs, though a gap remains between need and mental health service use. With limited knowledge of continued impact of mental health service use on future services and mental health need, this study aims to examine the longitudinal, bidirectional relations between mental health service utilization and mental health symptoms across four time points. Data came from four waves of the National Longitudinal Study of Adolescent Health (Add Health; N = 7,902). We examined mental health service (MHS) utilization in the past 12 months and depressive symptoms. Cross-lagged panel analyses were conducted, adjusting for sociodemographic characteristics. Findings indicated that school MHS utilization at Tn increased the odds of using MHS at Tn+1 across all waves (OR = 1.34, 1.53, and 1.61, respectively). Second, depressive symptoms at Tn also predicted depressive symptoms at Tn+1 (β = .21, .30, .30, respectively). Third, as MHS use at Tn predicted depressive symptoms at Tn+1 (β = .14 (school), .27, .11, .18, respectively), depressive symptoms at Tn were significantly related to future mental health service use at Tn+1 (OR = 1.09, 1.15, 1.05 respectively). The current study extends the understanding of the reciprocal relationship between MHS use and depressive symptoms. The study suggests the critical importance of using school-based services in adolescence to promote mental health service use in adulthood, highlighting implications for adolescent mental health service providers across service settings.

 Adolescence is a formative time where individuals begin to experience biological, psychological, and social changes, increasing susceptibility to mental health concerns. ^1^ Studies have consistently found that mental health disorders and symptoms (including anxiety, depression, panic) are most often identified in adolescence ^2–5^. Approximately 49.5% of adolescents have had a mental disorder in their lifetime. ^6,7^ These mental health disorders and symptoms can often transition from adolescence to early adulthood. ^8,9^ Notably, depression may follow a recurrent course. In their longitudinal study, Kovacs and colleagues found that 72% of adolescents who experienced a major depressive episode had a second episode, underscoring the chronic nature of depression and the need for early and sustained intervention. ^10^ If left untreated, potential outcomes of mental health disorders, such as depression, in childhood and adolescence can lead to increased risks of substance use, poor academic and social functioning, and risk of suicidal behaviors. ^11,12^ In a systematic review examining the relationship between adult mental health outcomes and adolescent depression, Johnson and colleagues report that 17 of the 18 articles they included found that depression in adolescence increases risk of depression in adulthood. ^13^ Of these 17 articles, 11 found that risk of depression in adulthood increased nearly three times when adolescent depression was present. ^13^ Overall, a growing need for services exists among youth and adults, with mental health needs persisting across age groups. ^14–16^ A report by Mongelli et al. suggests there remains a gap in mental health care referring to the disparities in people with a disorder and those who receive care due to barriers such as, stigma, mental health shortages, lack of insurance coverage, and limited resources. ^17^ For instance, individuals with limited income or from low-income households have also been found to be less likely to seek treatment for mental health needs due to their inability to access services (Hodgkinson et al., 2017; Menas, 2019). ^18,19^ Additionally, stigma contributes to gender disparities, in particular women are more likely to seek mental health services and treatment than their male counterparts. ^20,21^ Furthermore, disparities such as race and ethnicity impact use of mental health services. For instance, research by Terlizzi & Norris found that Non-Hispanic White individuals are most likely to use mental health services, whereas Asian, Black, and Hispanic adults are reported to have much lower use of services in comparison ^21^. Timely access to mental health treatment and services has been associated with a reduced risk of future symptoms. ^2,22,23^ Goktan et al. found that use of mental health treatment was linked to less worry, nervousness, and moodiness. ^24^ Similarly, Collins & Munoz-Solomando found that from a sample of almost 100 individuals who sought mental health services during childhood and adolescence, over half recovered from their diagnosis by the time they reached adulthood. ^2^ Thus, necessitates the importance of closing existing gaps in mental health services to prevent persistent mental health symptoms through adolescent to adulthood. Closing mental health service gaps, particularly for adolescents, is being addressed by implementing mental health supports within schools. This decreases several barriers that students face from utilizing services, such as socioeconomic status and transportation. ^20,25^ Schools are becoming a primary location for youth to access mental health services. Studies over the last decade have indicated that students who seek services have a higher likelihood to receive them in their school. ^26,27^ A recent study found that adolescents from low-income households have increasingly accessing services from their schools over the last several years. ^28^ Schools continue to be a critical source for early identification of mental health symptoms and for initial access to services. ^29,30^

## Current Study

As increasing rates of mental health needs among adolescents and adults continue, understanding service needs and utilization across time is becoming more pertinent. Additionally, though research suggests the importance of school-based mental health service use, there is a lack of understanding of future service use from adolescence to adulthood compared to school mental health services during adolescence. In particular, considering the continuing need for mental health services and the increasing mental health symptoms we are seeing among adolescents and adults, ^31,32^ it is important to understand the longitudinal relationship between service need and use.

Guided by Andersen’s Behavioral Model, ^33^ the current study conducts a cross-lagged panel analysis to examine the reciprocal relationship between mental health service use and depressive symptoms. Cross-lagged panel analyses allow for the examination of reciprocal relationships between variables over multiple timepoints, offering insights into potential directional influences when randomization is not feasible, though they do not establish definitive causality. ^34,35^ This study aims to examine the longitudinal, reciprocal relations between mental health service utilization and mental health symptoms across four time points.

## Methods

### Data and Sample

The current study uses restricted data from Waves I, III, IV, and V of the National Longitudinal Study of Adolescent Health (Add Health). ^36^ Add Health is a school-based study conducted through the Carolina Population Center at the University of North Carolina. Add Health is a longitudinal study of over 20,000 adolescents beginning in 7th through 12th grade and has followed participants through five waves. Wave I data (T1) was collected from September 1994 to April 1995 using in-school questionnaires. Wave III (T2) data collection aimed to follow up with T1 participants from the in-home survey as they transitioned into adulthood and was collected from August 2001 to April 2002 (ages 18–26), completing interviews with 15,170 participants (76% response rate). Wave IV (T3) data (participants aged 24–32) were collected across the USA between January 2008 and February 2009 with a response rate of 80.3%, with a total of 15,701 participants. ^37^ Wave V (T4) was conducted between 2016 and 2018 and utilized a mixed-mode survey, with online and mail surveys with in-person and phone non-response follow-ups, reaching 12,297 participants.

Participants were between the ages of 32 and 42 at the time of T4 data collection. Participants for the current study were limited to those who indicated attending school and being in grades 7–12 and between 12 and 17 years old during T1 of data collection. In accordance with using the WLSMV estimator (see analysis plan below) ^38^ only participants who responded to all four waves of data were included in the current study for a final sample of 7902. ^39^ It is important to note that Wave II of data was not included in this study because during this collection period, some participants were still in high school and some were note, so it did not support the research question relation to school-based mental health service use.

### Measures

#### Mental Health Service Use

Mental health service use was defined as having received a psychological or emotional counseling within the last 12 months. At each survey timepoint, participants were asked whether and where they had used such services in the last 12 months. School-based mental health services were measured at Time 1 (T1), during adolescence- a period when caregivers often play a central role in facilitating service access. In this context, service use at later waves (T2–T4) reflects a broader measure of mental health service engagement in adulthood, when individuals are more autonomous. This variable was a primary predictor in the analysis, with other types of service use (i.e., school) at T1 included as a covariate. The focus on school-based services reflects evidence supporting the importance of early intervention for long-term mental health. ^40^ Participants who indicated receiving school-based mental health services were coded as 1 (yes) and those who did not were coded as 0 (no). To account for mental health services in adulthood, participants were assessed at T2, T3, and T4 whether they had received psychological or emotional counseling in the past 12 months. Responses were coded as 1 (yes) if the services were received in any setting (e.g., private doctor’s office, community health clinic, school, hospital, or other), and 0 (no) if they had not received services.

#### Depressive Symptoms

Depressive symptoms were measured at each timepoint with three consistent items from the 20-item Center for Epidemiological Studies-Depression (CES-D), ^41^ as not all items were present for each wave of data collection. ^42^ Participants were asked how often they experienced each item in the last week (“you felt depressed,” “you felt sad,” “you felt that you could not shake the blues”) on a 4-point Likert scale from 0 (= never/rarely) to 3 (= most of the time). Items were reverse scored when necessary to indicate higher scores are higher levels of depressive symptoms and summed across the three items, ranging from 0 to 9. Reliability was acceptable at T1 (*⍺* = 0.79), and good at T2 (*⍺* = 0.81), T3 (*⍺* = 0.81), and T4 (*⍺* = 0.85).

#### Covariates

The current study included covariates based on the Expanded Behavioral Model of Health Care Utilization and its predicted factors that impact service utilization. ^33^ Age (in years), race/ethnicity, sex (0 = male, 1 = female), household income (time varying), and health insurance status (time varying) were included. There were six categories for race/ethnicity: non-Hispanic Asian/Pacific Islander, Non-Hispanic Black, non-Hispanic White, non-Hispanic Other races (including American Indian and Native American), non-Hispanic multiple races, and Hispanic. Household income at each timepoint was categorized into three groups: missing income, low resources (below median income), and high resources (median income and above), using the median household income of each year of data collection.A total of 43–46 Insurance status at each time point: no insurance or missing (= 0) and any form of insurance present (= 1).

#### Analysis Plan

Descriptive analysis was conducted in SPSS 28. In order to test bidirectional relationships to build evidence of causal relationships further, we examined the cross-lagged relations between mental health service utilization and depressive symptoms. Cross-lagged panel analyses will capitalize on the longitudinal nature of these data, allowing for causal inferences regarding the relationship between mental health service use setting and mental health symptoms. ^47^ The study adjusted for age, race/ethnicity, sex, and time varying household income and insurance status. Longitudinal data analyses were conducted in Mplus 8.7 using the WLSMV estimator with weighted data. The statistical significance was assessed at the 0.05 alpha level.

## Results

### Descriptive Statistics

Table 1 shows descriptive results, indicating that the majority of the sample identified as Non-Hispanic White (67.6%), female (66.6%), and the average age at T1 was 15.07 (*SD* = 1.47). Most participants (78.2 at T1, 78.0% at T2, 81.3% at T3, 91.1% at T4) had health insurance.

**Table 1:** Descriptive statistics for cross-lagged panel analysis sample (*N* = 7902)

Variables	Mean (SD) or %			
	Time 1	Time 2	Time 3	Time 4
Age (time 1)	15.07 (1.47)	–	–	–
Sex				
* Male*	33.4	–	–	–
* Female*	66.6	–	–	–
Race and Ethnicity				
* Non-Hispanic Asian/Pacific Islander*	5.3	–	–	–
* Non-Hispanic Black*	18.7	–	–	–
* Non-Hispanic Other Races*	1.2	–	–	–
* Non-Hispanic White*	67.6	–	–	–
* Non-Hispanic Bi or Multiracial*	3.8	–	–	–
* Hispanic*	13.5	–	–	–
Household income				
Missing	20.8	18.1	5.7	1.3
Low resources	30.9	62.5	40.3	54.8
High resources	48.4	19.3	53.9	43.8
Health insurance (yes)	78.2	78.0	81.3	91.1
School mental health service use (T1)	3.5	–	–	–
Other mental health service use	7.4	7.2	9.5	14.8
Depressive symptoms	1.45 (1.66)	1.18 (1.68)	1.22 (1.63)	1.32 (1.78)

Almost half (45.2%) of participants lived in a household classified as middle income at T1. At T2, the majority identified as having below median income (62.5%), more than median income at T3 (53.9%), and less than median income at T4 (54.8%). At T1, 3.5% of the sample used school-based mental health services, with 7.4% indicating use of other mental health services in the community. Service use increased at other timepoints, with 7.2% at T2, 9.5% at T3, and 14.8% at T4.

### Cross-Lagged Panel Analysis

**Figure 1:**
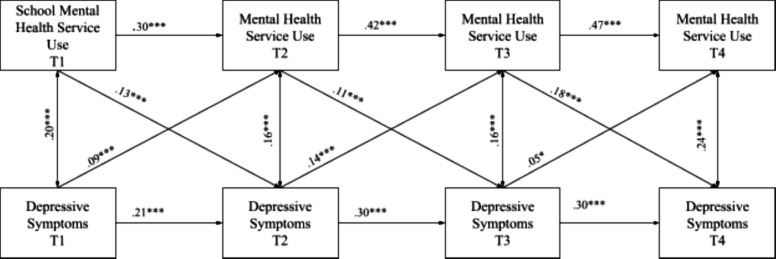
Cross-lagged relationships between mental health service utilization and depressive symptoms

Table 2 and Fig. 1 present the findings from the cross-lagged panel analysis with constant and time varying and covariates controlled for. The model had adequate model fit to the observed data (*χ*^2^ = 505.51 df = 90; *p* < 0.001, comparative fit index [CFI] = 0.87, Tucker-Lewis index [TLI] = 0.72, root mean square error of approximation [RMSEA] = 0.02). ^48^

**Table 2:** Direct effects for cross-lagged panel model (*N* = 7902)

		DV at time 1	DV at time 2	DV at time 3	DV at time 4
Dependent variable	Independent variable	Estimate (SE)	Estimate (SE)	Estimate (SE)	Estimate (SE)
Mental health service use	T1 use of other mental health services	**–**	.46 (.09)***	–	–
	T1 school mental health service use	**–**	.30 (.05)***	–	–
	T2 mental health service use	–	–	.42 (.04)***	–
	T3 mental health service use	–	–	–	.47(.03)***
	T1 depressive symptoms	.20(.03)***	.09 (.03)**	–	–
	T2 depressive symptoms	–	.16 (.03)***	.14 (.03)***	–
	T3 depressive symptoms	–	–	.16 (.03)***	.05 (.02)*
	T4 depressive symptoms	–	–	**–**	.24 (.02)***
	Age	** − **.02 (.04)	.001 (.03)	** − **.006 (.03)	.000 (.03)
	Sex (female)	.21 (.08)**)	.20 (.07)**	.14 (.07)*	.10 (.06)
	Race/ethnicity (ref = White) Non-Hispanic Asian/Pacific Islander Non-Hispanic Black Non-Hispanic Other Races Non-Hispanic Bi or Multiracial Hispanic	.34 (.23) .07 (.10) .33 (.23) .17 (.20) .26 (.11)*	−.45 (.28) −.27 (.10)** −.06 (23) −.03 (.19) −.29 (.11)**	−.43 (.20)* −.30 (.09)** −.01 (.24) −.009 (.18) −.13 (.10)	.02 (.15) −.002 (.07) .13 (.23) .08 (.12) .008 (.09)
	T1 household income (ref = high resource) Missing Low resources	** − **.12 (.11) .01 (.09)	– –	– –	– –
	T2 household income (ref = high resource) Missing Low resources	– –	.06(.09) .000 (.07)	**–** **–**	– –
	T3 household income (ref = high resource) Missing Low resources	– –	– –	.08 (.12) .12 (.06)*	– –
	T4 household income (ref = high resource) Missing Low resources	– –	– –	– –	.14 (.20) ** − **.04 (.06)
	T1 health insurance	** − **.13 (.09)	–	–	**–**
	T2 health insurance	–	** − **.07 (.07)	–	–
	T3 health insurance	–	–	.15 (.08)	–
	T4 health insurance	–	–	–	.24 (.09)**
Depressive symptoms	T1 use of other mental health services	–	.27 (.06)***	–	–
	T1 school mental health service use	.20(.03)***	.13 (.03)***	–	–
	T2 mental health service use	–	.16 (.03)***	.11 (.02)***	–
	T3 mental health service use	–	–	.16 (.03)***	.18 (.02)***
	T4 mental health service use				.24 (.02)***
	T1 depressive symptoms	–	.21 (.01)***	–	–
	T2 depressive symptoms	–	–	.30 (.01)***	–
	T3 depressive symptoms	–	–	–	.30 (.01)***
	Age	.13(.02)***	−.05 (.02)**	.002 (.02)	.02 (.02)
	Sex (female)	.33(.03)***	.23 (.04)***	.10 (.04)**	.02 (.04)
	Race/ethnicity (ref = White) Non-Hispanic Asian/Pacific Islander Non-Hispanic Black Non-Hispanic Other Races Non-Hispanic Bi or Multiracial Hispanic	.29(.80)*** .08 (.04) .17 (.12) .23(.08)** .17(.05)***	−.08 (.11) .06 (.04) .02 (.19) −.03 (.08) −.003 (.05)	.11 (.10) .16 (.04)***.23 (.11)* .18 (.09) .03 (.06)	−.02 (.11) −.04 (.05) −.13 (.18) .15 (.07)* .02 (.05)
	T1 household income (ref = high resource) Missing Low resources	.06 (.05) .08(.04)*	– –	– –	– –
	T2 household income (ref = high resource) Missing Low Income	– –	08 (.05) .03 (.05)	– –	– –
	T3 household income (ref = high resource) Missing Low Income			.38 (.06)*** .25 (.04)***	
	T4 household income (ref = high resource) Missing Low income				.21 (.12) .21 (.04)***
	T1 health insurance	** − **.02 (.04)			
	T2 health insurance		−.14 (.04)***		
	T3 health insurance			−.22 (.04)***	
	T4 health insurance				−.26 (.05)***

### Mental Health Service Use

As shown in Fig. 1, when looking at mental health services as the outcome, individuals who used school mental health services at T1 had 34% higher odds of using mental health services at T2 compared to those who did not (OR = 1.34, *p* < 0.001), and those who used other mental health services at T1 had 58% higher odds of using services at T2 (OR = 1.58, *p* < 0.001), use of services at T2 significantly increased the odds of using services at T3 (OR = 1.53, *p* < 0.001), and use of services at T3 significantly increased the odds of service use at T4 (OR = 1.61, *p* < 0.001). Increased depressive symptoms as T1 increased the odds of mental health service use at T2 (OR = 1.09, *p* = 0.001), with increased symptoms at T2 increasing odds of service use at T3 (OR = 1.15, *p* < 0.001), and increased symptoms at T3 increasing the odds of service use at T4 (OR = 1.05, *p* = 0.044).

Identifying as female increased the odds of service use at T1 (OR = 1.23, *p* < 0.001), T2 (OR = 1.22, *p* = 0.004), and T3 (OR = 1.15, *p* = 0.031). Participants who identified as Hispanic were 30% more likely to use mental health services at T1 compared to their NonHispanic White counterparts (OR = 1.30, *p* = 0.014). Conversely, identifying as Black (OR = 0.76, *p* = 0.005) or Hispanic (OR = 0.75, *p* = 0.008) decreased the odds of service use at T2, while identifying as Asian/Pacific Islander (OR = 0.65, *p* = 0.029) or Black decreased the odds of using mental health services at T3 (OR = 0.74, *p* = 0.001). Having low-income resources at T3 was associated with increased odds of using services at T3 (OR = 1.13, *p* = 0.04), and having health insurance at T4 increased one’s odds of using services at T4 (OR = 1.27, *p* = 0.006).

#### Depressive Symptoms

School mental health service use at T1 (OR = 1.14, *p* < 0.001), as well as other mental health service use (OR = 1.31 *p* < 0.001), predicted increased depressive symptoms at T2. Use of mental health services at T2 was associated with increased depressive symptoms at T3 (OR = 1.12, *p* < 0.001), and service use at T3 was associated with increased symptoms at T4 (OR = 1.20, *p* < 0.001). Additionally, increased depressive symptoms at T1 was associated with increased symptoms at T2 (OR = 1.23, *p* < 0.001), T2 with T3 (OR = 1.35, *p* < 0.001), and T3 with T4 (OR = 1.35, *p* < 0.001).

Older participants had increased depressive symptoms at T1 (OR = 1.14, *p* < 0.001) and decreased depressive symptoms at T2 (OR = 0.95, *p* = 0.009). Identifying as female was associated with increased depressive symptoms at T1 (OR = 1.39, *p* < 0.001), T2 (OR = 1.26, *p* < 0.001), and T3 (OR = 1.11, *p* = 0.004). NonHispanic Asian/Pacific Islander (OR = 1.34, *p* < 0.001), bi- or multiracial (OR = 1.26, *p* = 0.003), and Hispanic (OR = 1.19, *p* < 0.001) participants all had increased depressive symptoms at T1. Identifying as NonHispanic Black (OR = 1.17, *p* < 0.001) and NonHispanic Other Races (OR = 1.26, *p* = 0.038) were associated with increased depressive symptoms at T3, while identifying as biracial or multiracial was associated with increased symptoms at T4 (OR = 1.16, *p* = 0.038). Those with low-income resources (compared to high-income resources were associated with increased depressive symptoms at T1 (OR = 1.08 *p* = 0.044), T3 (OR = 1.28, *p* < 0.001), and T4 (OR = 1.23, *p* < 0.001). Additionally, participants with health insurance had decreased symptoms of depression at T2 (OR = 0.87, *p* < 0.001), T3 (OR = 0.80, *p* < 0.001), and T4 (OR = 0.77, *p* < 0.001).

## Discussion

The current study extends research on mental health service use and depressive symptoms by examining cross-lagged relationships among these two primary variables. This study aimed to increase the understanding of the reciprocal relationship between the two and to assess the temporal ordering and bidirectional associations between variables.

The current study found that the use of mental health services increased the odds of subsequent service use. Specifically, use of school-based, as well as other services, was a significant predictor of continued service use at T2, during young adulthood. To the authors’ knowledge, no study has examined how school-based mental health service use impacts future service use, which was one of the aims of the current study. These findings are especially relevant in light of the high rates of recurrent depression among adolescents. Given that a significant proportion of youth who experience depressive symptoms go on to experience multiple episodes (Kovacs et al., 2016), early contact with school-based mental health services may support not only short-term coping but also ongoing engagement with care during high-risk developmental periods. Munson et al. reported the importance of consistent and continued treatment for mental health needs for young adults. ^49^ Adolescence is a time of many developmental changes, including shifts in decision-making for their own care and health and new systems of care as they transition to adult services. ^50,51^ While the study findings underscore the potential of school-based services to influence long-term service utilization, it is important to acknowledge that quality, type, and availability of services varies across schools and states. Recent data suggests that only 39% of schools provide family-level interventions, 34% provide outreach services, and 17% of states provide telehealth services. ^32^ This variation may impact the effectiveness and continuity of care for youth. Therefore, efforts to enhance consistency and accessibility are essential to ensure that early engagement in mental health services leads to sustained service use into adulthood.

After adjusting for age, race/ethnicity, sex, household family income, and insurance at each timepoint, the model consistently found that increased depressive symptoms increased the likelihood of using mental health services at each timepoint. These findings support existing research and theoretical frameworks that suggest increased mental health needs lead to increased service utilization. ^33,52–54^ For example, one study found that for adults with mental health diagnoses, perceived need, including distress, emotional problems, and externalizing behaviors, led to increased rates of service utilization. Knowing that increased mental health need is linked to increased rates of service use, it is important at all ages for individuals to have increased knowledge of what services and treatments are available to them and how they can access such services. The current study also found that females were more likely to seek mental health services, while minoritized races were less likely than their non-Hispanic White counterparts. The disproportionate utilization of mental health services by individuals of color is well documented, particularly for Black individuals. ^55,56^ Several studies link such underutilization to the negative stigma associated with mental health in communities of color. ^57–59^ Finding ways to reduce such stigma is paramount as needs for services continue to increase.

The model also found that the use of mental health services predicted subsequent increased depressive symptoms for all timepoints. This was an interesting and important finding, as research to date has had mixed results. Studies have suggested how mental health treatment is beneficial for individuals, decreasing their symptoms or recovering from diagnoses over time. ^22^ While this is true, studies have also found that previous use of mental health treatment is linked to increased depressive symptoms. ^60,61^ This finding may be linked to previous studies indicating that previous use of services or continued use of services could indicate increased levels of symptoms and severity of need, ^62^ which does link to literature suggesting elevated mental health needs during young adulthood. ^63^ Additionally, such discrepancy between treatment and symptoms could be due to quality of care. Jorm et al. suggested that while research often hypothesizes that closing the gap between need and treatment should decrease mental health needs, the quality of care may be the barrier to seeing a reduction in prevalence of mental health disorders and symptoms. ^64^ Further, this could also be attributed to mental health stigma; as more individuals recognize symptoms of mental health disorders, this may increase contact with providers and diagnosis. ^64^ Such findings are important for practice and policy. As mental health needs continue to increase following the impact of COVID-19, it is critical that knowledge of mental health symptoms increases for individuals to recognize when they may need to seek services.

### Limitations

In light of the important findings from this study, there are several limitations. Depressive symptoms were measured using the CES-D, ^41^ but different questions were used at each timepoint. ^42^ Due to the limited number of consistent questions regarding depressive symptoms, only four indicators were used in the analyses. Studies utilizing longitudinal data collection should consider items that are consistently used in each wave of data collection to support improved measurement of constructs. Add Health was also limited in other mental health symptom domains that were included across waves of data. Additional symptoms, including those for anxiety, could add deeper understanding of mental health symptoms and service use over time. Future studies should examine additional barriers and facilitators to service use and predictors of need. It is also important to acknowledge the omission of variables related to trauma. Though these important contextual variables were not present in the current model, future research should include trauma and age of onset of mental health symptoms due to their relationship with more severe trajectories of symptoms. ^5,11^

An additional limitation of this study is the inability to contextualize how mental health services and treatment were defined. While the Add Health data contextualized the location of service delivery when asking about utilization, respondents were not prompted regarding what specific services they received in any setting (i.e., therapy, medication, and management). This is a limitation due to the secondary nature of data for the current study. Future studies should further delve into the context of what specific services and treatments individuals received, as this would lead to very important implications for mental health policy and practice.

In addition, the distribution of several variables was a limitation of the study and led to collapsing categories further than desired. Among all participants, use of mental health services was very low, with a mere 3.5% of participants using school-based mental health services at T1. Though this is a limitation, this is the reality of service utilization and an important implication when considering the need for increased service use. There were also high numbers of missing cases for household income as well as uneven distribution. To not lose a substantial number of participants due to missing values, missing for household income was included. Categories for income options were also collapsed in order to account for distribution issues, limiting the full examination of different income levels.

## Implications for Behavioral Health

Findings concur with previous studies indicating that using mental health services is linked to increased depressive symptoms. This study reinforces Andersen’s framework that need is a driving factor for why individuals seek mental health services, indicating a critical need to continue closing the gap between service need and service use through continued efforts to improve quality of service, increase knowledge of mental health needs and how to access services, and decrease the stigma of service use. Findings highlight the continued impact of early engagement with mental health services, particularly school-based services, on continued utilization in adulthood. This suggests the importance of strengthening early identification and intervention strategies as well as supports for sustaining long-term engagement. Findings also point to several important directions for future inquiry. Future research is needed to examine barriers to service use and mental health symptoms while considering different facilitators that may support service use among different populations. Continued research regarding service location (ex. school and community) could be beneficial for this critical area of research.

### Compliance of Interest Statement

There are no conflicts of interest for this study. The study utilized secondary data from the National Longitudinal Study of Adolescent Health; therefore, the current study did not include research involving human subjects and was exempt from IRB review at the authors’ institution. Due to the use of secondary data, information regarding informed consent can be retrieved via the National Longitudinal Study of Adolescent Health methodologies guidebook.

## Data Availability

Data for the current study was from restricted-use data from the National Longitudinal Study of Adolescent to Adult Health. Public-use data is available to the public.

## Declarations

### The National Longitudinal Study of Adolescent to Adult Health

Public-use data is available to the public.
